# p57 Suppresses the Pluripotency and Proliferation of Mouse Embryonic Stem Cells by Positively Regulating p53 Activation

**DOI:** 10.1155/2021/4968649

**Published:** 2021-12-24

**Authors:** Na Li, Zhaoyu Du, Yunxiang Li, Wenjing Xu, Yumei Yang, Haodong Peng, Tianxiang Song, Qihua Qin, Huining Lei, Jinlian Hua

**Affiliations:** College of Veterinary Medicine, Shaanxi Center of Stem Cells Engineering & Technology, Northwest A & F University, Yangling, 712100 Shaanxi, China

## Abstract

Embryonic stem cells (ESCs) are pluripotent stem cells that have indefinite self-renewal capacities under appropriate culture conditions *in vitro*. The pluripotency maintenance and proliferation of these cells are delicately governed by the concert effect of a complex transcriptional regulatory network. Herein, we discovered that p57^Kip2^ (p57), a cyclin-dependent kinase inhibitor canonically inhibiting cell proliferation, played a role in suppressing the pluripotency state of mouse ESCs (mESCs). p57 knockdown significantly stimulated the expressions of core pluripotency factors NANOG, OCT4, and SOX2, while p57 overexpression inhibited the expressions of these factors in mESCs. In addition, consistent with its function in somatic cells, p57 suppressed mESC proliferation. Further analysis showed that p57 could interact with and contribute to the activation of p53 in mESCs. In conclusion, the present study showed that p57 could antagonize the pluripotency state and the proliferation process of mESCs. This finding uncovers a novel function of p57 and provides new evidence for elucidating the complex regulatory of network of mESC fate.

## 1. Introduction

Embryonic stem cells (ESCs) are pluripotent cells derived from the inner cell mass of a preimplantation blastocyst [1]. These cells are characterized by an indefinite self-renewal capacity and pluripotency, with the potential to differentiate into cells of all three germ layers [1]. The self-renewal and pluripotency of ESCs are delicately modulated by a variety of internal and external signals and governed by a concert effect of the transcriptional regulatory network [2]. Among the transcriptional factors studied, NANOG, OCT4, and SOX2 are at the heart of the network to maintain the self-renewal and pluripotency of ESCs [3]. In addition, other proteins such as LIF, Klf4, Tbx3, Otx2, p53, and Foxo1/3a also critically regulate ESC fate [4–7]. In order to fully identify the clinical potential of ESCs, it is pivotal to understand how ESC fate is controlled by the intricate regulatory network and whether other unknown signaling proteins/pathways are involved in this network.

p57^Kip2^ (p57) belongs to the Cip/Kip family that could block cell proliferation by inhibiting the activities of cyclins and cyclin-dependent kinases (CDK) [8]. This canonical function of the cyclin/CDK inhibitor p57 is well-established and has been extensively reported. p57 can bind to all cyclins and CDKs and functions as an ATP mimic, thereby preventing the binding of ATP with these cell cycle regulation proteins [9, 10]. In addition, p57 has a proliferating cell nuclear antigen- (PCNA-) binding domain through which its interaction with PCNA prevents PCNA-dependent DNA replication [11]. By doing so, p57 blocks cells in the G1 phase and inhibits cell cycle progression. Indeed, downregulation of p57 expression usually accelerates cell proliferation and this is frequently observed during the development of many cancers, making p57 an important tumor suppressor [8, 10, 12].

More recently, emerging evidence has identified and characterized a variety of novel functions for p57 in addition to its role in cell cycle regulation. For example, p57 plays an important role in determining the differentiation process of several cell types. p57-null mice exhibited numerous and severe abnormalities in cell proliferation and differentiation, characterized by cleft palate, abdominal muscle formation defects, endochondral bone ossification delay and bone shortening, adrenal hyperplasia, renal dysplasia, and lens cell hyperproliferation and apoptosis [13, 14]. A reduction in p57 expression was observed in parallel with delayed chondrocyte differentiation [15]. During skeletal muscle differentiation, the suppression of p57 expression resulted in abortive myoblast differentiation, while induction of p57 efficiently restored this differentiation process [16]. Other studies also showed that the expression profile of p57 determined neurogenesis via cell differentiation regulation of the central and peripheral nervous systems [17, 18].

In comparison, the role of p57 in stem cell modulation is relatively unclear. Several studies showed that p57 was required for maintaining the quiescence state of hematopoietic stem cells (HSCs), hair follicle stem cells, and neural stem cells [18–20]. In addition, proper p57 expression is necessary for the self-renewal of bronchioalveolar stem cells [21]. p57 expression was also observed in a subset of intestinal stem cells, although its function was not explored in this study [22]. Studies regarding the role of p57 in ESCs are far less. There is limited evidence indicating that p57 is involved in affecting ESC proliferation as a downstream signaling protein, while direct evidence is lacking [23, 24]. In addition, whether p57 plays a role in pluripotency maintenance of ESCs remains undetermined.

In this study, we provide evidence showing that p57 acted to suppress the pluripotency and proliferation of mESCs, and this effect was mediated through a positive modulation of p53 activation. Our findings uncover a novel connection between p57 and the self-renewal of mESCs.

## 2. Materials and Methods

### 2.1. Culture of mESCs

mESCs were purchased from ATCC (SCRC-1010, Manassas, USA) and cultured on mouse embryonic fibroblast feeder layer and maintained in high-glucose Dulbecco's Modified Eagle Medium (DMEM) (Gibco, Waltham, Massachusetts, USA) supplemented with 15% FBS (Gibco), 10 ng/mL leukemia inhibitory factor (LIF) (Sino Biological, Beijing, China), 0.1 mmol/L *β*-mercaptoethanol (Sigma-Aldrich, St. Louis, Missouri, United States), and 1% nonessential amino acids (Gibco). mESCs were passaged using TrypLE™ Select (Gibco) at a ratio of 1 : 8 every 2 days.

### 2.2. Retinoic Acid (RA) Treatment

1 × 10^5^ mESCs were plated into each well of 12-well plates and cultured in the above mESC medium. For RA treatment, each well of the cultured mESCs was treated by 2 *μ*M of RA (Sigma-Aldrich) in DMSO or DMSO alone of the same volume for 48 h.

### 2.3. Embryoid Body (EB) Formation and Differentiation

2 × 10^6^ mESCs were suspended in a 35 mm nonadherent culture dish (Axygen Biotechnology, Hangzhou, China) in mESC medium described above. Two days later, EBs were formed. The EBs were further transferred into 12-well plates in mESC medium without LIF for another 3 days to allow their spontaneous differentiation.

### 2.4. Real-Time Quantitative PCR

Total RNA was extracted with TRIzol Reagent (Takara, Kusatsu, Japan), and reverse transcription was performed using a RevertAid RT Reverse Transcription Kit (Thermo Fisher Scientific, Waltham, Massachusetts, USA). Real-time PCR analysis was conducted using a SYBR Premix Ex Taq II Kit (Takara). Data were collected using a Bio-Rad CFX96 system (Bio-Rad, Hercules, California, USA). The primers used in real-time PCR are listed in Table S1. The reference gene used for real-time PCR data analysis was GAPDH in this article.

### 2.5. Western Blot

Western blot was performed as we previously reported [25]. The antibodies used in this article are listed as follows: anti-p57 (1 : 500, Cell Signaling Technology, Danvers, Massachusetts, USA), anti-p53 (1 : 500, Wanleibio, Xi'an, China), anti-p-p53 (1 : 500, Wanleibio, Shenyang, China), anti-GAPDH (1 : 5000; Genesci, Beijing, China), anti-PCNA (1 : 1000, Boster, Wuhan, China), anti-Cyclin A (1 : 300, Santa Cruz, Dallas, Texas, USA), anti-Cyclin E (1 : 300, Santa Cruz), anti-OCT4 (1 : 500, Santa Cruz), anti-NANOG (1 : 500, PeproTech, Rocky Hill, New Jersey, USA), anti-SOX2 (1 : 1000, Proteintech Group, Rosemont, Illinois, USA), horse-radish peroxidase-conjugated anti-rabbit antibody (1 : 3000, Boster), and anti-mouse antibody (1 : 2000; Boster).

### 2.6. EdU Staining

EdU staining was conducted using a Cell-Light EdU Apollo 567 In Vitro Kit (Ribobio, Guangzhou, China) as previously described [26]. For the counting of EdU-positive cells, at least 3 fields of cells were randomly chosen and the percentage of EdU-positive cells of each field was counted. The mean value of the fields was calculated as the final percentage of EdU-positive cells.

### 2.7. Alkaline Phosphatase (AP) Staining

AP staining was performed using AST Fast Red TR and *α*-Naphthol AS-MX Phosphate (Sigma-Aldrich) according to the manufacturer's instructions.

### 2.8. Coimmunoprecipitation Assay

The coimmunoprecipitation assay was performed as we previously described [27]. Information for the antibodies used is listed as follows: anti-Flag (1 : 1000, Sigma-Aldrich), anti-p53 (1 : 1000, Wanleibio), horse-radish peroxidase-conjugated anti-rabbit antibody (1 : 3000; Boster), and anti-mouse antibody (1 : 2000; Boster).

### 2.9. p57 Knockdown, Overexpression, Cell Transfection, and Treatment with p53 Inhibitor

For p57 knockdown, a recombinant plasmid containing short-hairpin RNA against p57 (shp57), namely, the pSIH1-H1-shp57-CoGFP plasmid, was constructed as previously reported [27]. Shp57 fragment was designed as CTTAAGTGCGCATTTTTGGTGTGTAAGTAGAAGTCAATTGATCATATTGACTTCTACTTACACACCCCTAGG, as indicated in Figure S1.

For p57 overexpression, p57 primer (Table S2) was designed using primer 5. p57 gene was obtained by PCR amplification according to the instructions of PrimeStar Max Premix (Takara) and analyzed by agarose gel electrophoresis. The target band was harvested using a TIANgel Midi Purification Kit (Tiangen, Beijing, China). The collected p57 gene and PCDH-EF1-3×FLAG-T2A-Puro plasmid were treated using QuickCut restriction enzyme (Takara) according to the manufacturer's instructions and further linked together using T4 DNA Ligase (Takara) according to the manufacturer's instructions.

Lentivirus packaging and cell infection were performed as previously described [28]. In brief, pSIH1-H1-shp57-CoGFP plasmid or PCDH-EF1-3×FLAG-p57-T2A-Puro (or the corresponding empty plasmid pSIH1-H1-CoGFP or PCDH-EF1-3×FLAG-T2A-Puro) was transfected with PAX and VSV-G into 293 T cells using TurboFect Transfection Reagent (Thermo Fisher Scientific) according to the manufacturer's instructions. The cell culture medium was replaced with fresh medium after 12 h of culture. The cell culture supernatant was harvested after another 48 h and then mixed with mESC culture medium at 1 : 1 containing 10 *μ*g/mL of polybrene (Sigma-Aldrich) to infect mESCs. 12 h later, the transfection medium was replaced with fresh mESC culture medium and the mESCs were harvested after another 48 h for further analyses.

mESCs overexpressing PCDH-EF1-3×FLAG-p57-T2A-Puro and PCDH-EF1-3×FLAG-T2A-Puro were treated with 10 *μ*M/mL of pifithrin-*α* hydrobromide (MedChemExpress, Monmouth Junction, NJ, USA) for 48 h and subjected to further analyses.

### 2.10. Bimolecular Fluorescence Complementation (BiFC) Assay

Primers for *p57*, *p53*, *Pcna*, *p21*, *p27*, *p16*, *Wnt6*, and *Wnt2* were designed using primer 5 and are listed in Table S2. For BiFC assay, *p57* gene was linked with pBiFC-VC155 (Addgene, Watertown, Massachusetts, USA), while *p53*, *Pcna*, *p21*, *p27*, *p16*, *Wnt6*, and *Wnt2* genes were linked with pBiFC-VN173 (Addgene) as described above. Before transfection, 1 × 10^5^ 293 T cells were plated into each well of 12-well plates. BiFC-*p57*-VC155 was cotransfected with BiFC-*p53*-VN173 (or BiFC-*Pcna*-VN173, BiFC-*p21*-VN173, BiFC-*p27*-VN173, BiFC-*p16*-VN173, BiFC-*Wnt6*-VN173, and BiFC-*Wnt2*-VN173) using TurboFect Transfection Reagent (Thermo Fisher Scientific) according the manufacturer's instructions. 6 h later, fresh culture medium was changed and the cells were further cultured for 48 h before analysis.

### 2.11. Cell Apoptosis Assay *via* Flow Cytometry

Cell apoptosis was analyzed by an Annexin V-FITC/PI apoptosis kit (Multi Sciences, Hangzhou, China) as previously described [29]. The samples were tested by a flow cytometer (BD Biosciences, USA) and analyzed using FlowJo software.

### 2.12. Statistical Analysis

All assays were replicated for at least 3 times in the present study. Data are presented as mean ± standard deviation. Statistical significance was determined using two-tailed Student's *t*-test. Difference was considered significantly if the calculated *P* value was less than 0.05 (^∗^*P* < 0.05, ^∗∗^*P* < 0.01, and ^∗∗∗^*P* < 0.001).

## 3. Results

### 3.1. Increased p57 Expression during mESC Differentiation

**Figure 1:**
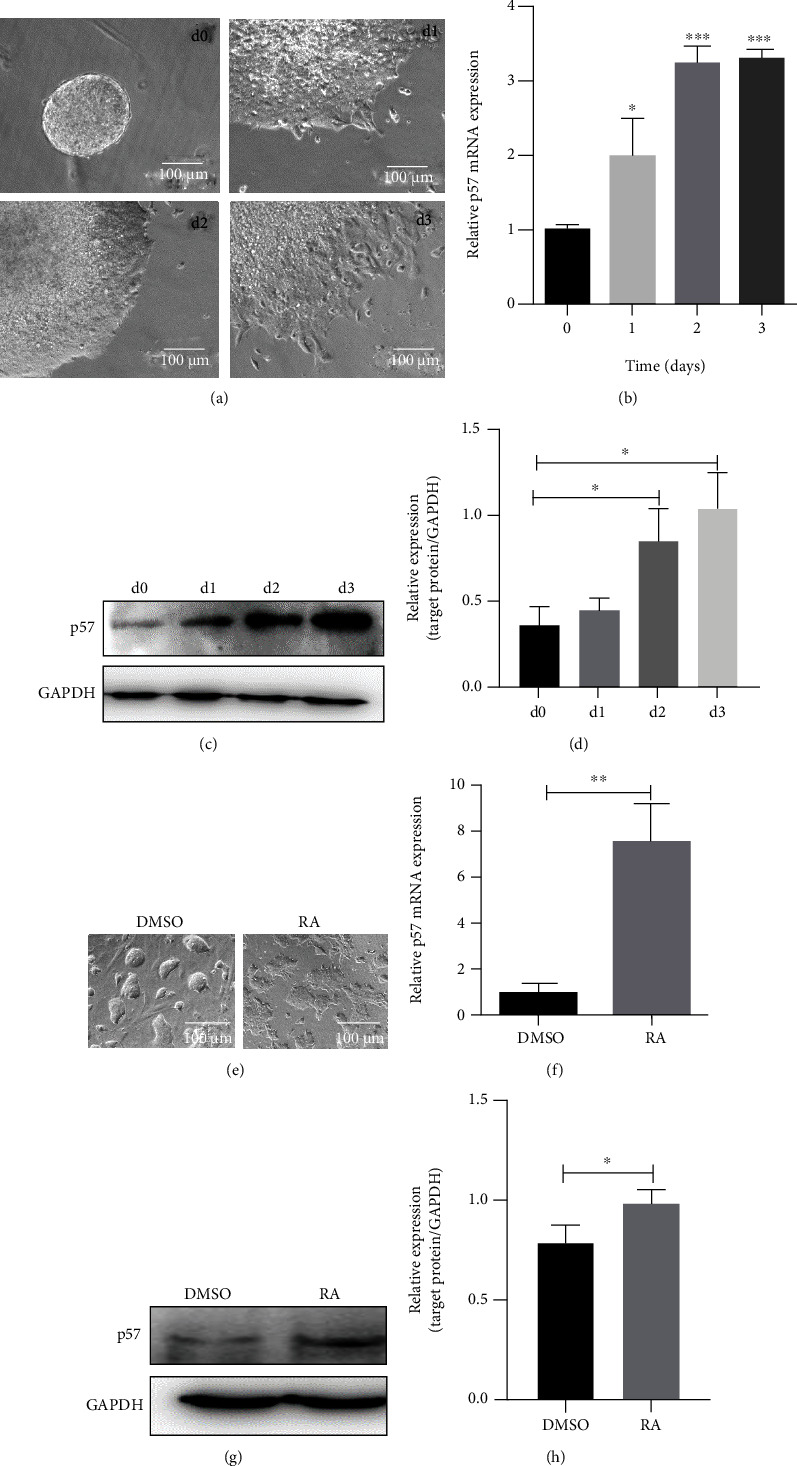
Increased p57 expression during mESC differentiation. (a) The morphology of mESC-derived embryoid bodies (EBs) cultured for 3 days. (b) Real-time PCR analysis of p57 expression level during EB differentiation from day 0 to day 3 (*n* = 3). (c, d) Western blot and related densitometric analysis of p57 expression level during EB differentiation from day 0 to day 3. (e) The morphology of mESCs treated with DMSO or RA for 48 h. (f) Real-time PCR analysis of p57 expression level of DMSO- or RA-treated mESC (*n* = 3 for both DMSO and RA-treated mESCs). (g, h) Western blot and related densitometric analysis of p57 expression level of DMSO- or RA-treated mESCs. Scale bar = 100 *μ*m. ^∗^*P* < 0.05, ^∗∗^*P* < 0.01, and ^∗∗∗^*P* < 0.001.

To investigate the role of p57 in mESCs, its expression level was analyzed in undifferentiated and differentiated mESCs. During the spontaneous differentiation of mESC-derived embryoid bodies (EBs), the expression level of p57 increased significantly (Figures 1(a)–1(d), Figure S7A). RA is one of the most effective inducers of mESC differentiation. During RA-induced differentiation of mESCs, higher p57 expression was also observed (Figures 1(e)–1(h), Figure S7B).

### 3.2. p57 Acted to Suppress the Pluripotency State of mESCs

**Figure 2:**
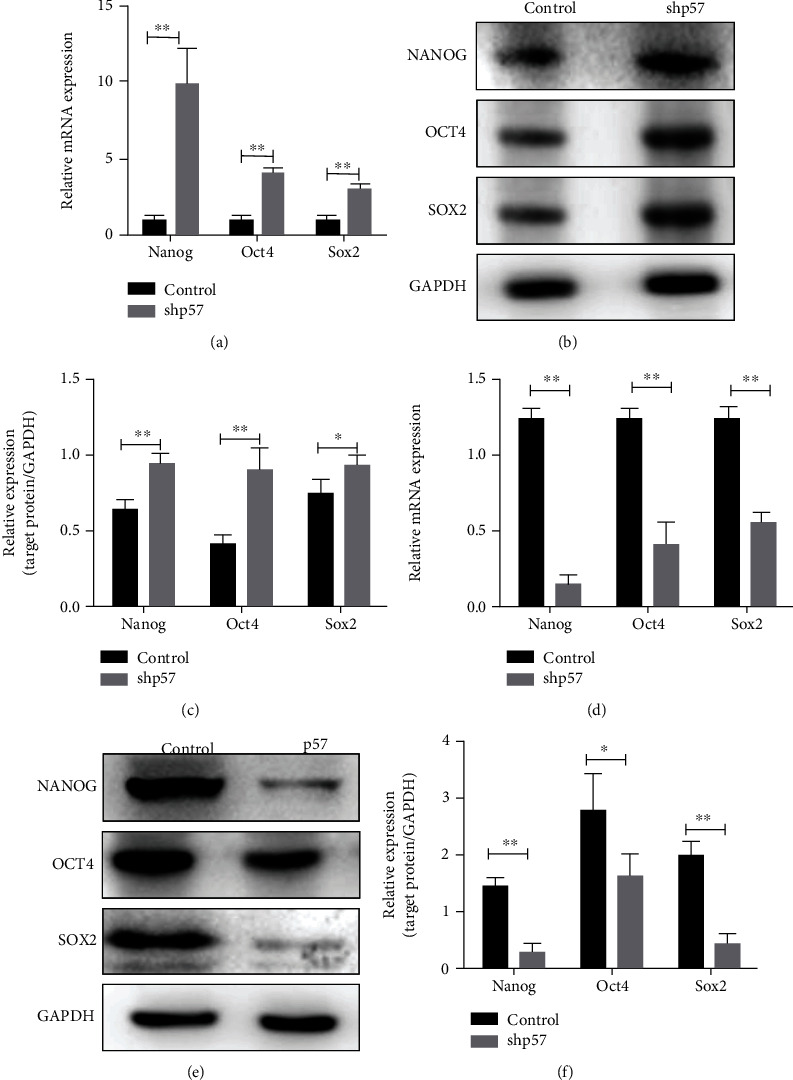
p57 suppressed the pluripotency state of mESCs. (a) Real-time PCR analyses of *Nanog*, *Oct4*, and *Sox2* expression levels in control (*n* = 3) and p57 knockdown (shp57, *n* = 3) mESCs. (b, c) Western blot and related densitometric analyses of NANOG, OCT4, and SOX2 protein levels in control and p57 knockdown (shp57) mESCs cultured for 48 h. (d) Real-time PCR analyses of *Nanog*, *Oct4*, and *Sox2* expression levels in control (*n* = 3) and p57-overexpressing (p57, *n* = 3) mESCs cultured for 48 h. (e, f) Western blot and related densitometric analyses of NANOG, OCT4, and SOX2 protein levels in control and p57-overexpressing (p57) mESCs cultured for 48 h. ^∗^*P* < 0.05, ^∗∗^*P* < 0.01, and ^∗∗∗^*P* < 0.001.

The increased expression of p57 during mESC differentiation suggested that p57 may potentially affect the pluripotency maintenance of mESCs. This idea prompted us to further examine the effects of p57 knockdown and overexpression in mESCs. The efficiencies for p57 knockdown and overexpression were confirmed at both mRNA and protein levels (Figures S1 and S2, Figure S11A-B). In response to p57 interference, mESCs expressed significantly higher levels of NANOG, OCT4, and SOX2, all essential transcription factors to maintain the pluripotent state of mESCs (Figures 2(a)–2(c), Figure S8A). In line with these results, p57 overexpression inhibited the expression of NANOG, OCT4, and SOX2 (Figures 2(d)–2(f), Figure S8B). The suppressing effect of p57 on the pluripotency state of mESCs seems to be long-lasting, as this suppression was also evident after 2, 4, and 6 days of culture (Figure S6).

### 3.3. p57 Suppressed the Proliferation of mESCs

**Figure 3:**
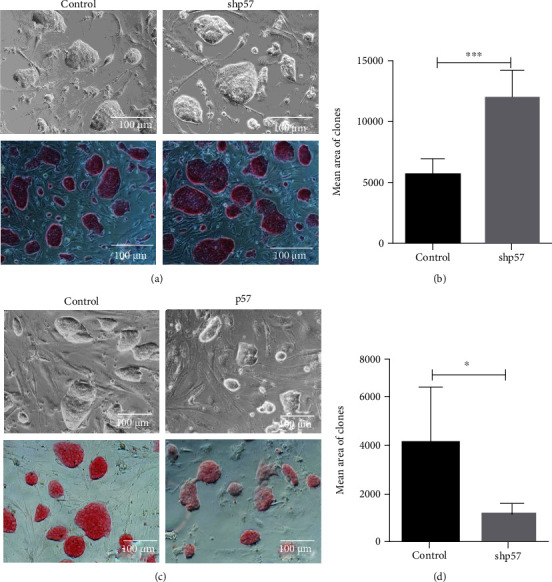
p57 suppressed the clone areas of mESCs *in vitro*. (a) Representative image of morphology and AP expression profiles of control and p57 knockdown (shp57) mESCs cultured for 48 h. (b) Area analysis of clones of control (*n* = 5) and p57 knockdown (shp57, *n* = 5) mESCs. (c) Representative image of morphology and AP expression profiles of control and p57-overexpressing (p57) mESCs cultured for 48 h. (d) Area analysis of clones of control (*n* = 5) and p57-overexpressing (p57, *n* = 5) mESCs. Scale bar = 100 *μ*m. ^∗^*P* < 0.05 and ^∗∗∗^*P* < 0.001.

**Figure 4:**
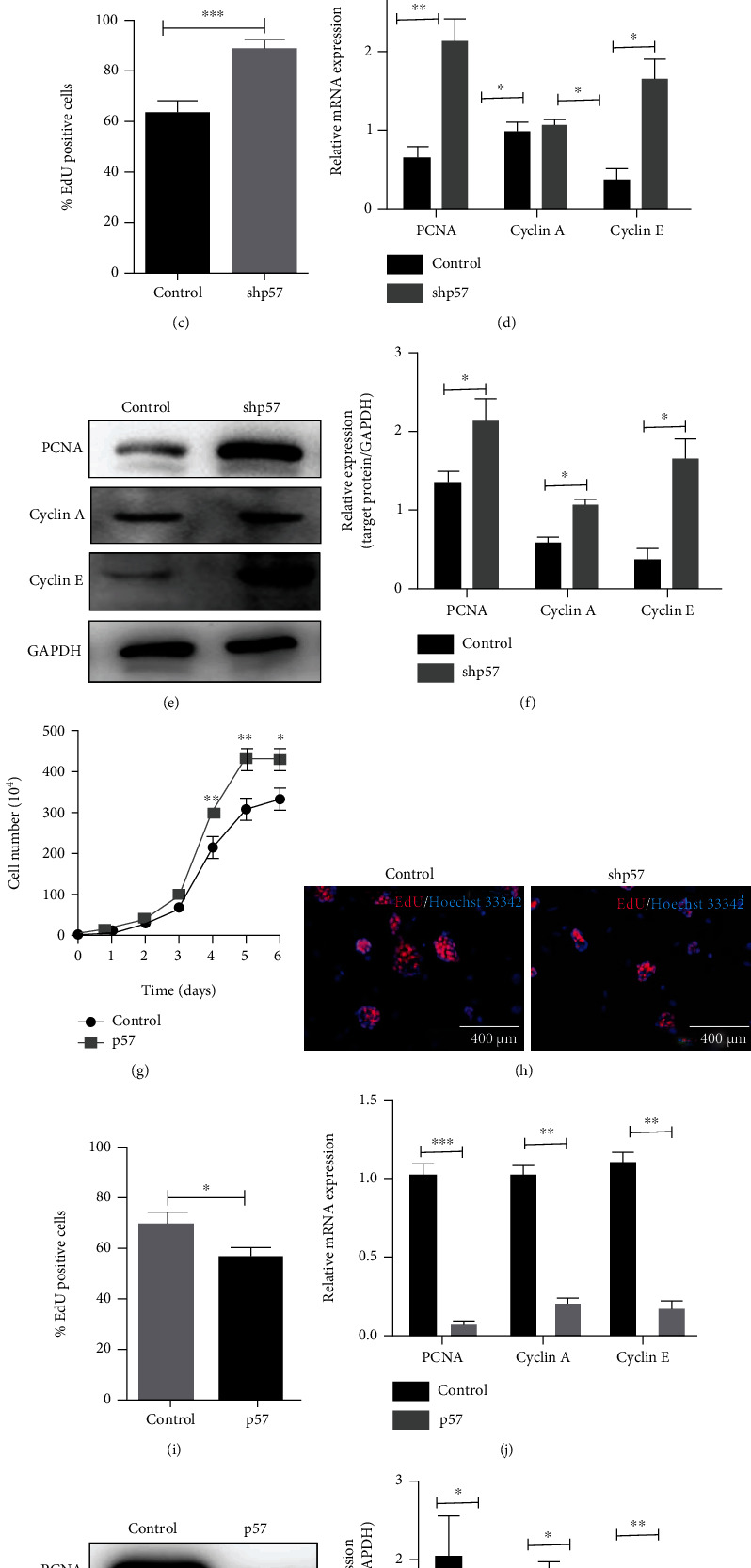
p57 suppressed the proliferation of mESCs. (a) The growth curve of control (*n* = 5) and p57 knockdown (shp57, *n* = 5) mESCs. (b, c) EdU staining results of control (*n* = 5) and p57 knockdown (shp57, *n* = 5) mESCs cultured for 48 h. (d) Real-time PCR analyses of *pcna*, *Cyclin A*, and *Cyclin E* expression levels of control (*n* = 3) and p57 knockdown (shp57, *n* = 3) mESCs cultured for 48 h. (e, f) Western blot and related densitometric analysis of PCNA, Cyclin A, and Cyclin E protein levels of control and p57 knockdown (shp57) mESCs cultured for 48 h. (g) The growth curve of control (*n* = 5) and p57-overexpressing (p57, *n* = 5) mESCs. (h, i) EdU staining results of control (*n* = 5) and p57-overexpressing (p57, *n* = 5) mESCs cultured for 48 h. (j) Real-time PCR analyses of *pcna*, *Cyclin A*, and *Cyclin E* expression levels of control (*n* = 3) and p57-overexpressing (p57, *n* = 3) mESCs cultured for 48 h. (k, l) Western blot and related densitometric analysis of PCNA, Cyclin A, and Cyclin E protein levels of control and p57-overexpressing (p57) mESCs cultured for 48 h. Scale bar = 400 *μ*m. ^∗^*P* < 0.05, ^∗∗^*P* < 0.01, and ^∗∗∗^*P* < 0.001.

Interestingly, in addition to affecting the pluripotent state of mESCs, we observed that p57 expression profile also influenced the areas of mESC clones. The result showed that p57 interference resulted in larger mESC clones while its overexpression produced much smaller mESC clones (Figures 3(a)–3(d)). This observation implicated that p57 could also restrain the proliferation of mESCs. To validate this assumption, we performed proliferation-associated analyses in mESCs. As expected, mESCs with p57 interference showed more vigorous proliferation rate, as reflected by higher cell numbers and greater EdU-incorporation abilities (Figures 4(a)–4(c)). In line with these observations, PCNA, Cyclin A, and Cyclin E, factors that are crucial for cell cycle progression, were also significantly upregulated in mESCs with p57 interference (Figures 4(d)–4(f), Figure S9A). Consistent with these findings, mESCs with p57 overexpression exhibited much lower proliferation ability and expressed reduced levels of proliferation-associated markers (Figures 4(g)–4(l), Figure S9B). The suppressing effect of p57 on the proliferation of mESCs seems to be long-lasting, as this suppression was also evident after 2, 4, and 6 days of culture (Figure S6). However, p57 has little effects on the apoptosis of mESCs (Figure S3A-B).

### 3.4. p57 Interacted with and Contributed to the Activations of p53 in mESCs

**Figure 5:**
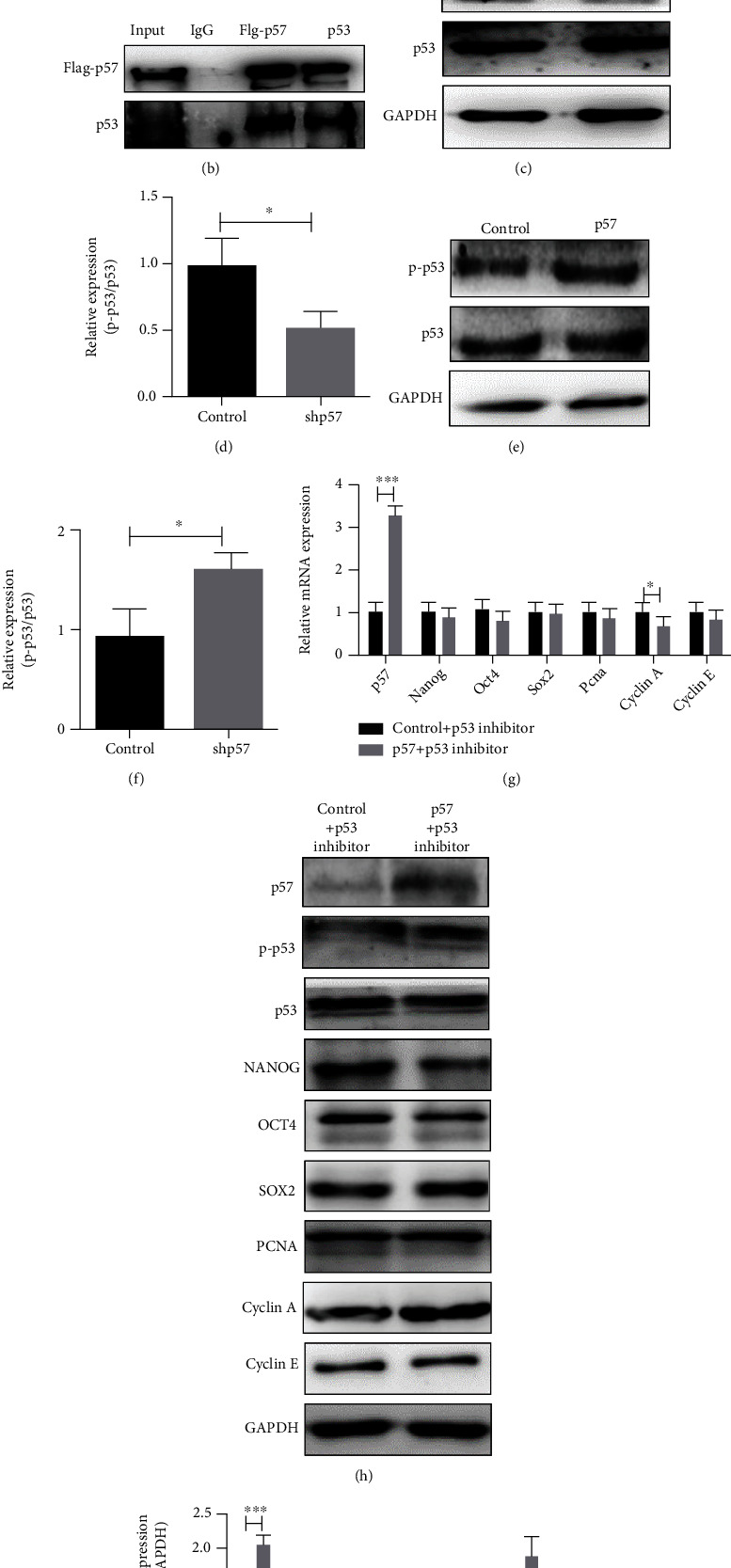
p57 interacted with and contributed to the activations of p53 in mESCs. (a) Visualization of p57-p53 interaction *in vivo* by BiFC assay (*n* = 3). (b) Coimmunoprecipitation of p57 (Flag-p57) and p53. (c, d) Western blot and related densitometric analysis of p53 and its phosphorylation level at Ser 315 in control and p57 knockdown (shp57) mESCs. (e, f) Western blot and related densitometric analysis of p53 and its phosphorylation level at Ser 315 in control and p57-overexpressing (p57) mESCs. (g) Real-time PCR analyses of *p57*, *Nanog*, *Oct4*, *Sox2*, *Pcna*, *Cyclin A*, and *Cyclin E* expression levels of control (*n* = 3) and p57-overexpressing (p57, *n* = 3) mESCs treated with p53 inhibitor cultured for 48 h. (h, i) Western blot and related densitometric analysis of p57, p-p53, p53, NANOG, OCT4, SOX2, PCNA, Cyclin A, and Cyclin E expression levels of control and p57-overexpressing (p57) mESCs treated with p53 inhibitor cultured for 48 h. Scale bar = 200 *μ*m. ^∗^*P* < 0.05.

The above evidence suggested that p57 played an important role in controlling the pluripotency and proliferation of mESCs. To further explore the underlying mechanism, we screened proteins interacting with p57 in mESCs. BiFC assays and coimmunoprecipitation assays both showed an active interaction between p57 and p53 (Figures 5(a) and 5(b), Figure S4, Figure S5). Further evidence revealed that although p57 did not affect the total expression level of p53, it could positively regulate the activation of p53, as p57 knockdown restrained while its overexpression promoted the phosphorylation of p53 at Ser 315, a phosphorylation site closely associated with p53 transcriptional activity [30] (Figures 5(c)–5(f), Figure S10A-B). As expected, under the action of p53 inhibitor pifithrin-*α* hydrobromide, the pluripotency- and proliferation-associated genes and proteins showed no significant changes between the p57 group and control group (Figures 5(g)–5(i), Figure S10C). Thus, the evidence presented here strongly suggested that the inhibition of mESC self-renewal by p57 is mediated, at least in part, by positive regulation of p53 activation.

## 4. Discussion

ESCs have the potential to differentiate into any type of terminal-differentiated somatic cells such as hepatocytes, cardiomyocytes, skeletal muscle cells, epithelial and vascular smooth muscle cells, neurons, and germ cells upon proper *in vitro* induction [25, 31, 32]. This distinguishing characteristic of ESCs has encouraged many attempts to employ human ESCs for the treatment of corresponding clinical problems such as end-stage liver diseases, heart failure, severe skin burns, stroke, Parkinson's disease, and infertility [33–38]. In order to fulfill the clinical application of human ESCs in regenerative medicine, basic researches that could provide a better understanding of the regulatory network governing the self-renewal and pluripotency of ESCs are necessary. In the present study, we identified p57 as a novel regulator of mESC pluripotency and proliferation, and we also demonstrated that the regulation of mESC by p57 was partly mediated via p53 signaling. These findings provided new evidence for elucidating the complex regulatory network of the fate of ESCs.

p57 is a pleiotropic protein that is involved in many important processes of various cell types. Its canonical role in blocking cell cycle progression as a cyclin/CDK repressor has been widely reported. The involvement of this protein in promoting neural precursor migration [39], stimulating neurogenesis, and promoting the differentiation process of chondrocytes and myoblasts has also been reported [15, 18, 32]. In stem cells, p57 mainly functions to maintain the quiescence state of these cells in several tissues. p57 are highly expressed in quiescent adult hematopoietic stem cells (HSC) and profoundly control the quiescence and stemness these cells [20, 40]. In mice with p57 deletion specifically in the hematopoietic system, decreased HSC pool with profoundly reduced self-renewal capacity was observed, which was caused by a failure of quiescence maintenance and increased apoptosis rate of these cells [20, 40]. Similarly, quiescent neural stem cells expressed high levels of p57 while proliferative progenitors exhibited very weak or completely undetectable p57 signals [18]. Further evidence suggested that p57 also regulated the quiescence state of neural stem cells and control the pace of lifelong neurogenesis [18]. In bronchioalveolar stem cells, either knockdown or overexpression of p57 caused defective self-renewal, which ultimately resulted in compromised lung regeneration after injury [21]. Unlike these adult stem cells that usually reside in the G0/G1 phase of the cell cycle, ESCs are characterized by fast proliferation and a short G1 phase, and this is closely associated with the pluripotency of ESCs [41]. However, studies regarding the role of p57 in embryonic stem cells are very limited. The elegant work of Li et al. showed that p57 was posttranscriptionally inhibited by microRNA miR-221 in mouse mESCs, making miR-221 critically required for mESC proliferation [42]. Similarly, p57 was a predicted target of an ESC-enriched miR-92b, which affected the G1 to S phase transition in human ESCs [23]. In addition, p57 was presumably involved in the regulation of human ESC proliferation by protein arginine methyltransferase 5 (PRMT5) [24]. Nevertheless, whether this protein plays a role in ESC pluripotency regulation remains unclear. The present study provides direct evidence showing that p57 antagonizes mESC pluripotency and this protein also functions to restrain mESC proliferation. Although it is unknown whether the effect of p57 on mESC pluripotency was a result of inhibited proliferation of mESC or a direct effect of p57, the evidence provided here unraveled a novel function of p57 and the underlying mechanisms deserve further in-depth investigations in the future.

The critical role of p53 in maintaining genomic stability as a tumor suppressor has been extensively reported and well-established in multiple somatic cells [43]. However, its role in ESCs remains much more elusive and has received great interest in the past few years. It has been shown that p53 activation stimulated the differentiation of ESCs by directly suppressing the genes required for ESC pluripotency. For example, p53 could bind to the promoter of *Nanog* and suppress its expression in response to DNA damage [30]. In line with this finding, Lee et al. found that a majority of p53-targeted genes in mESCs are involved in developmental processes, especially genes associated with mesodermal and ectodermal development [7]. Interestingly, p53 is not only able to induce ESC differentiation but also antagonizes the pluripotency and self-renewal of ESCs, a process involved with the activation of *miR-34a* and *miR-145*, two microRNAs potently repressing the expressions of Klf4, Oct4, Lin28a, and Sox2 [44]. Thus, it is not surprising that p53 activity is stringently regulated to guarantee ESC identity and fate. One regulator for p53 activity is Oct4, which prevents p53 activation *via* Sirt1-mediated deacetylation of p53 [45]. Findings of the present study again underscore the importance of p53 regulation in ESCs. To our knowledge, this is the first report describing the control of p53 by p57 in mESCs. However, further investigations are still needed to identify the detailed mechanism on how this regulation process works.

## 5. Conclusions

In the present study, we found that p57 knockdown promoted the expressions of core factors associated with mESC pluripotency, while its overexpression inhibited the expressions of these factors. In addition, p57 also suppressed the proliferation of mESCs. Further evidence showed that the function of p57 in mESCs was mediated by p53. Thus, p57 could negatively regulate the pluripotency state and the proliferation of mESCs through p53 activation.

## Data Availability

All data is available from the corresponding author Jinlian Hua (jinlianhua@nwsuaf.edu.cn) upon request.

## Supplementary Materials

Supplementary Materials Table S1: primer sequences used for real-time quantitative PCR. Table S2: primer sequences used for PCR amplification in BiFC assay. Figure S1: construction of shp57- and p57-overexpressing vectors. Figure S2: p57 knockdown or overexpression efficiency in ESCs. Figure S3: p57 have no effects on apoptosis of mESCs. Figure S4: construction of the vectors for BiFC assay. Figure S5: visualization of the interactions between p57 and candidate proteins (PCNA, p21, p27, p16, WNT6, and WNT2) *in vivo* by BiFC assay. Figure S6: the effect of p57 on mESCs at day 2, day 4, and day 6. Figure S7: increased p57 protein expression during mESC differentiation. Figure S8: p57 suppressed the pluripotency state of mESCs. Figure S9: p57 suppressed the proliferation of mESCs. Figure S10: p57 interacted with and contributed to the activations of p53 in mESCs. Figure S11: p57 knockdown or overexpression efficiency in mESCs.

## References

[1] Hassani S. N., Moradi S., Taleahmad S., Braun T., Baharvand H. (2019). Transition of inner cell mass to embryonic stem cells: mechanisms, facts, and hypotheses. *Cellular and molecular life sciences*.

[2] Yeo J. C., Ng H. H. (2013). The transcriptional regulation of pluripotency. *Cell Research*.

[3] Rodda D. J., Chew J. L., Lim L. H. (2005). Transcriptional regulation of nanog by OCT4 and SOX2. *The Journal of Biological Chemistry*.

[4] Acampora D., Di Giovannantonio L. G., Simeone A. (2013). Otx2 is an intrinsic determinant of the embryonic stem cell state and is required for transition to a stable epiblast stem cell condition. *Development*.

[5] Hall J., Guo G., Wray J. (2009). Oct4 and LIF/Stat3 additively induce Kruppel factors to sustain embryonic stem cell self-renewal. *Cell Stem Cell*.

[6] Esmailpour T., Huang T. S. (2012). TBX3 promotes human embryonic stem cell proliferation and neuroepithelial differentiation in a differentiation stage-dependent manner. *Stem Cells*.

[7] Lee D. F., Su J., Ang Y. S. (2012). Regulation of embryonic and induced pluripotency by aurora kinase-p53 signaling. *Cell Stem Cell*.

[8] Creff J., Besson A. (2020). Functional versatility of the CDK inhibitor p57(Kip2). *Frontiers in Cell and Developmental Biology*.

[9] Russo A. A., Jeffrey P. D., Patten A. K., Massague J., Pavletich N. P. (1996). Crystal structure of the p27Kip1 cyclin-dependent-kinase inhibitor bound to the cyclin A-Cdk2 complex. *Nature*.

[10] Matsuoka S., Edwards M. C., Bai C. (1995). p57KIP2, a structurally distinct member of the p21CIP1 Cdk inhibitor family, is a candidate tumor suppressor gene. *Genes & Development*.

[11] Watanabe H., Pan Z. Q., Schreiber-Agus N., DePinho R. A., Hurwitz J., Xiong Y. (1998). Suppression of cell transformation by the cyclin-dependent kinase inhibitor p57KIP2 requires binding to proliferating cell nuclear antigen. *Proceedings of the National Academy of Sciences of the United States of America*.

[12] Guo H., Jing L., Cheng Y. (2016). Down-regulation of the cyclin-dependent kinase inhibitor p57 is mediated by Jab1/Csn5 in hepatocarcinogenesis. *Hepatology*.

[13] Zhang P. M., Liegeois N. J., Wong C. (1997). Altered cell differentiation and proliferation in mice lacking p57(KIP2) indicates a role in Beckwith-Wiedemann syndrome. *Nature*.

[14] Yan Y., Frisen J., Lee M. H., Massague J., Barbacid M. (1997). Ablation of the CDK inhibitor p57(Kip2) results in increased apoptosis and delayed differentiation during mouse development. *Genes & Development*.

[15] Simsa-Maziel S., Monsonego-Ornan E. (2012). Interleukin-1 beta promotes proliferation and inhibits differentiation of chondrocytes through a mechanism involving down-regulation of FGFR-3 and p21. *Endocrinology*.

[16] Roeb W., Boyer A., Cavenee W. K., Arden K. C. (2007). PAX3-FOXO1 controls expression of the p57Kip2 cell-cycle regulator through degradation of EGR1. *Proceedings of the National Academy of Sciences of the United States of America*.

[17] Pfurr S., Chu Y. H., Bohrer C. (2017). The E2A splice variant E47 regulates the differentiation of projection neurons via p57(KIP2) during cortical development. *Development*.

[18] Furutachi S., Matsumoto A., Nakayama K. I., Gotoh Y. (2013). p57 controls adult neural stem cell quiescence and modulates the pace of lifelong neurogenesis. *The EMBO Journal*.

[19] Leishman E., Howard J. M., Garcia G. E. (2013). Foxp1 maintains hair follicle stem cell quiescence through regulation of Fgf18. *Development*.

[20] Matsumoto A., Takeishi S., Kanie T. (2011). p57 is required for quiescence and maintenance of adult hematopoietic stem cells. *Cell Stem Cell*.

[21] Zacharek S. J., Fillmore C. M., Lau A. N. (2011). Lung stem cell self-renewal relies on BMI1-dependent control of expression at imprinted loci. *Cell Stem Cell*.

[22] Barriga F. M., Montagni E., Mana M. (2017). Mex3a marks a slowly dividing subpopulation of Lgr5+intestinal stem cells. *Cell Stem Cell*.

[23] Sengupta S., Nie J., Wagner R. J., Yang C., Stewart R., Thomson J. A. (2009). MicroRNA 92b controls the G1/S checkpoint gene p57 in human embryonic stem cells. *Stem Cells*.

[24] Gkountela S., Li Z., Chin C. J., Lee S. A., Clark A. T. (2014). PRMT5 is required for human embryonic stem cell proliferation but not pluripotency. *Stem Cell Reviews and Reports*.

[25] Li N., Ma W., Shen Q. (2019). Reconstitution of male germline cell specification from mouse embryonic stem cells using defined factors in vitro. *Cell Death and Differentiation*.

[26] Zhu Z., Pan Q., Zhao W. (2021). BCL2 enhances survival of porcine pluripotent stem cells through promoting FGFR2. *Cell Proliferation*.

[27] Wei Y., Yang D., Du X. (2020). Interaction between DMRT1 and PLZF protein regulates self-renewal and proliferation in male germline stem cells. *Molecular and cellular biochemistry*.

[28] Wei Y. D., Du X. M., Yang D. H. (2021). Dmrt1 regulates the immune response by repressing the TLR4 signaling pathway in goat male germline stem cells. *Zoological Research*.

[29] Li B., He X., Zhuang M. (2018). Melatonin ameliorates busulfan-induced spermatogonial stem cell oxidative apoptosis in mouse testes. *Antioxidants & Redox Signaling*.

[30] Lin T., Chao C., Saito S. (2005). p53 induces differentiation of mouse embryonic stem cells by suppressing Nanog expression. *Nature Cell Biology*.

[31] Agarwal S., Holton K. L., Lanza R. (2008). Efficient differentiation of functional hepatocytes from human embryonic stem cells. *Stem Cells*.

[32] Guan K., Rohwedel J., Wobus A. M. (1999). Embryonic stem cell differentiation models: cardiogenesis, myogenesis, neurogenesis, epithelial and vascular smooth muscle cell differentiation in vitro. *Cytotechnology*.

[33] Wu D. B., Chen E. Q., Tang H. (2018). Stem cell transplantation for the treatment of end-stage liver disease. *World Journal of Hepatology*.

[34] Menasche P., Vanneaux V. (2016). Stem cells for the treatment of heart failure. *Current Research in Translational Medicine*.

[35] Li Y., Xia W. D., Van der Merwe L., Dai W. T., Lin C. (2020). Efficacy of stem cell therapy for burn wounds: a systematic review and meta-analysis of preclinical studies. *Stem Cell Research & Therapy*.

[36] Singh M., Pandey P. K., Bhasin A., Padma M. V., Mohanty S. (2020). Application of stem cells in stroke: a multifactorial approach. *Frontiers in Neuroscience*.

[37] Fan Y., Winanto N. S. Y. (2020). Replacing what's lost: a new era of stem cell therapy for Parkinson's disease. *Translational Neurodegeneration*.

[38] Lorzadeh N., Kazemirad N. (2018). Embryonic stem cells and infertility. *American Journal of Perinatology*.

[39] Tury A., Mairet-Coello G., DiCicco-Bloom E. (2011). The cyclin-dependent kinase inhibitor p57Kip2 regulates cell cycle exit, differentiation, and migration of embryonic cerebral cortical precursors. *Cerebral Cortex*.

[40] Zou P., Yoshihara H., Hosokawa K. (2011). p57(KiP2) and p27(Kip1) cooperate to maintain hematopoietic stem cell quiescence through interactions with Hsc70. *Cell Stem Cell*.

[41] Coronado D., Godet M., Bourillot P. Y. (2013). A short G1 phase is an intrinsic determinant of naive embryonic stem cell pluripotency. *Stem Cell Research*.

[42] Li J., Bei Y., Liu Q. (2015). MicroRNA-221 is required for proliferation of mouse embryonic stem cells via P57 targeting. *Stem Cell Reviews and Reports*.

[43] Sammons M. A., Nguyen T. A. T., McDade S. S., Fischer M. (2020). Tumor suppressor p53: from engaging DNA to target gene regulation. *Nucleic Acids Research*.

[44] Jain A. K., Allton K., Iacovino M. (2012). p53 regulates cell cycle and microRNAs to promote differentiation of human embryonic stem cells. *PLoS Biology*.

[45] Zhang Z. N., Chung S. K., Xu Z., Xu Y. (2014). Oct4 maintains the pluripotency of human embryonic stem cells by inactivating p53 through Sirt1-mediated deacetylation. *Stem Cells*.

