# Supplementary material for: p57 Suppresses the Pluripotency and Proliferation of Mouse Embryonic Stem Cells by Positively Regulating p53 Activation
Source: Stem Cells Int. 2021 Dec 24;2021:4968649. doi: 10.1155/2021/4968649 (PMC8720024; doi:10.1155/2021/4968649)
Supplement: Supplementary Materials — Table S1: primer sequences used for real-time quantitative PCR. Table S2: primer sequences used for PCR amplification in BiFC assay. Figure S1: construction of shp57- and p57-overexpressing vectors. Figure S2: p57 knockdown or overexpression efficiency in ESCs. Figure S3: p57 have no effects on apoptosis of mESCs. Figure S4: construction of the vectors for BiFC assay. Figure S5: visualization of the interactions between p57 and candidate proteins (PCNA, p21, p27, p16, WNT6, and WNT2) in vivo by BiFC assay. Figure S6: the effect of p57 on mESCs at day 2, day 4, and day 6. Figure S7: increased p57 protein expression during mESC differentiation. Figure S8: p57 suppressed the pluripotency state of mESCs. Figure S9: p57 suppressed the proliferation of mESCs. Figure S10: p57 interacted with and contributed to the activations of p53 in mESCs. Figure S11: p57 knockdown or overexpression efficiency in mESCs. [file 4968649.f1.zip › Supplementary Figure legends (1).docx]

**Supplementary Figure legends**

**Supplementary Figure 1 Construction of shp57 and p57-overexpressing vectors.** (A) Plasmid map of pSIH1-H1-*shp57*-CoGFP. (B) Plasmid map of PCDH-EF1-3×FLAG-*p57*-T2A-Puro. (C) Identification of PCDH-EF1-3×FLAG-*p57*-T2A-Puro with restriction enzyme.

**Supplementary Figure 2 p57 knockdown or overexpression efficiency in mESCs.** (A) Real-time PCR analyses of *p57* in control (n=3) and p57 knockdown (shp57, n=3). (B-C) Western blot and related densitometric analysis p57 protein level in control and shp57 group. (D) Real-time PCR analyses of *p57* in control (n=3) and p57-overexpressing (p57, n=3). (E-F) Western blot and related densitometric analysis p57 protein level in control and p57 group. n≥3 for each group, *P < 0.5, ** P < 0.01, *** P < 0.001.

**Supplementary Figure** 3 **p57 have no effects on apoptosis of mESCs.** (A) Apoptosis analysis of control (n=4) and p57 knockdown (shp57, n=4) mESCs using flow cytometry. (B) Apoptosis analysis of control (n=4) and p57-overexpressing (p57, n=4) mESCs using flow cytometry.

**Supplementary Figure** 4 **Construction of the vectors for BiFC assay.** (A) Identification of BiFC-*p57*-VC155 with restriction enzyme. (B) Identification of BiFC-*p53*-VN173 with restriction enzyme. (C) Identification of BiFC-*Pcna*-VN173 with restriction enzyme. (D) Identification of BiFC-*p21*-VN173with restriction enzyme. (E) Identification of BiFC-*p27*-VN173 with restriction enzyme. (F) Identification of BiFC-*p16*-VN173 with restriction enzyme. (G) Identification of BiFC-*Wnt6*-VN173 with restriction enzyme. (H) Identification of BiFC-*Wnt2*-VN173 with restriction enzyme.

**Supplementary Figure** 5 **Visualization of the interactions between p57 and candidate proteins (PCNA, p21, p27, p16, WNT6 and WNT2) *in vivo* by BiFC assay.** Scale bar = 200 μm.

**Supplementary Figure 6 The effect of p57 on mESCs at day2, day4 and day 6.** (A) Real-time PCR analyses of *p57*, *Nanog*, *Oct4*, *Sox2*, *Pcna,* *Cyclin A* and *Cyclin E* in control (n=3) and p57 knockdown (shp57, n=3) mESCs at day 2. (B) Real-time PCR analyses of *p57*, *Nanog*, *Oct4*, *Sox2*, *Pcna,* *Cyclin A* and *Cyclin E* in control (n=3) and p57 knockdown (shp57, n=3) mESCs at day 4. (C) Real-time PCR analyses of *p57*, *Nanog*, *Oct4*, *Sox2*, *Pcna,* *Cyclin A* and *Cyclin E* in control (n=3) and p57 knockdown (shp57, n=3) mESCs at day 6. (D) Real-time PCR analyses of *p57*, *Nanog*, *Oct4*, *Sox2*, *Pcna,* *Cyclin A* and *Cyclin E* in control and p57-overexpressing (p57, n=3) mESCs at day 2. (E) Real-time PCR analyses of *p57*, *Nanog*, *Oct4*, *Sox2*, *Pcna,* *Cyclin A* and *Cyclin E* in control and p57-overexpressing (p57, n=3) mESCs at day 4. (F) Real-time PCR analyses of *p57*, *Nanog*, *Oct4*, *Sox2*, *Pcna,* *Cyclin A* and *Cyclin E* in control and p57-overexpressing (p57, n=3) mESCs at day 6.

**Supplementary Figure 7 Increased p57 protein expression during mESC differentiation.** (A) Western blot analysis of p57 expression level during EB differentiation from day 0 to day 3. (B) Western blot analysis of p57 expression level of DMSO- or RA-treated mESCs.

**Supplementary Figure 8 p57 suppressed the pluripotency state of mESCs. (A)** Western blot analysis of NANOG, OCT4 and SOX2 protein levels in control and p57 knockdown (shp57) mESCs. (B) Western blot analysis of NANOG, OCT4 and SOX2 protein levels in control and p57-overexpressing (p57) mESCs.

**Supplementary Figure 9 p57 suppressed the proliferation of mESCs.** (A) Western blot analysis of PCNA, Cyclin A and Cyclin E protein levels of control and p57 knockdown (shp57) mESCs. (B) Western blot analysis of PCNA, Cyclin A and Cyclin E protein levels of control and p57-overexpressing (p57) mESCs.

**Supplementary Figure 10 p57 interacted with and contributed to the activations of p53 in mESCs.** (A) Western blot analysis of p53 and its phosphorylation level at Ser 315 in control and p57 knockdown (shp57) mESCs. (B) Western blot analysis of p53 and its phosphorylation level at Ser 315 in control and p57-overexpressing (p57) mESCs. (C) Western blot analysis of p57, p-p53, p53, NANOG, OCT4, SOX2, PCNA, Cyclin A and Cyclin E expression levels of control and p57-overexpressing (p57) mESCs treated with p53 inhibitor.

**Supplementary Figure 11 p57 knockdown or overexpression efficiency in mESCs.** (A) Western blot analysis p57 protein level in control and p57 knockdown (shp57) mESCs. (B) Western blot analysis p57 protein level in control and p57-overexpressing (p57) mESCs.
