# Supplementary material for: p57 Suppresses the Pluripotency and Proliferation of Mouse Embryonic Stem Cells by Positively Regulating p53 Activation
Source: Stem Cells Int. 2021 Dec 24;2021:4968649. doi: 10.1155/2021/4968649 (PMC8720024; doi:10.1155/2021/4968649)

Supplementary Figure 1

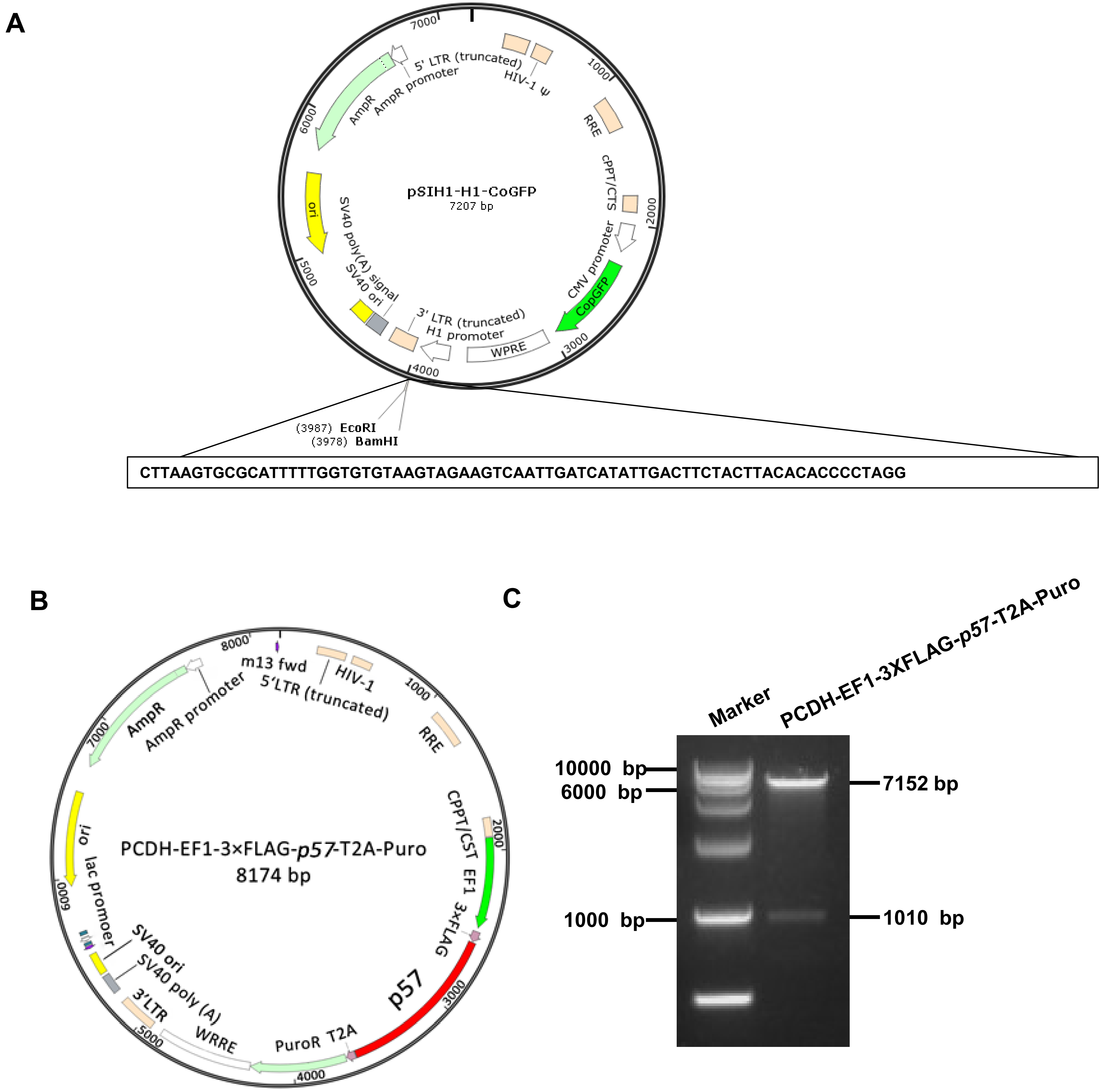

Supplementary Figure 2

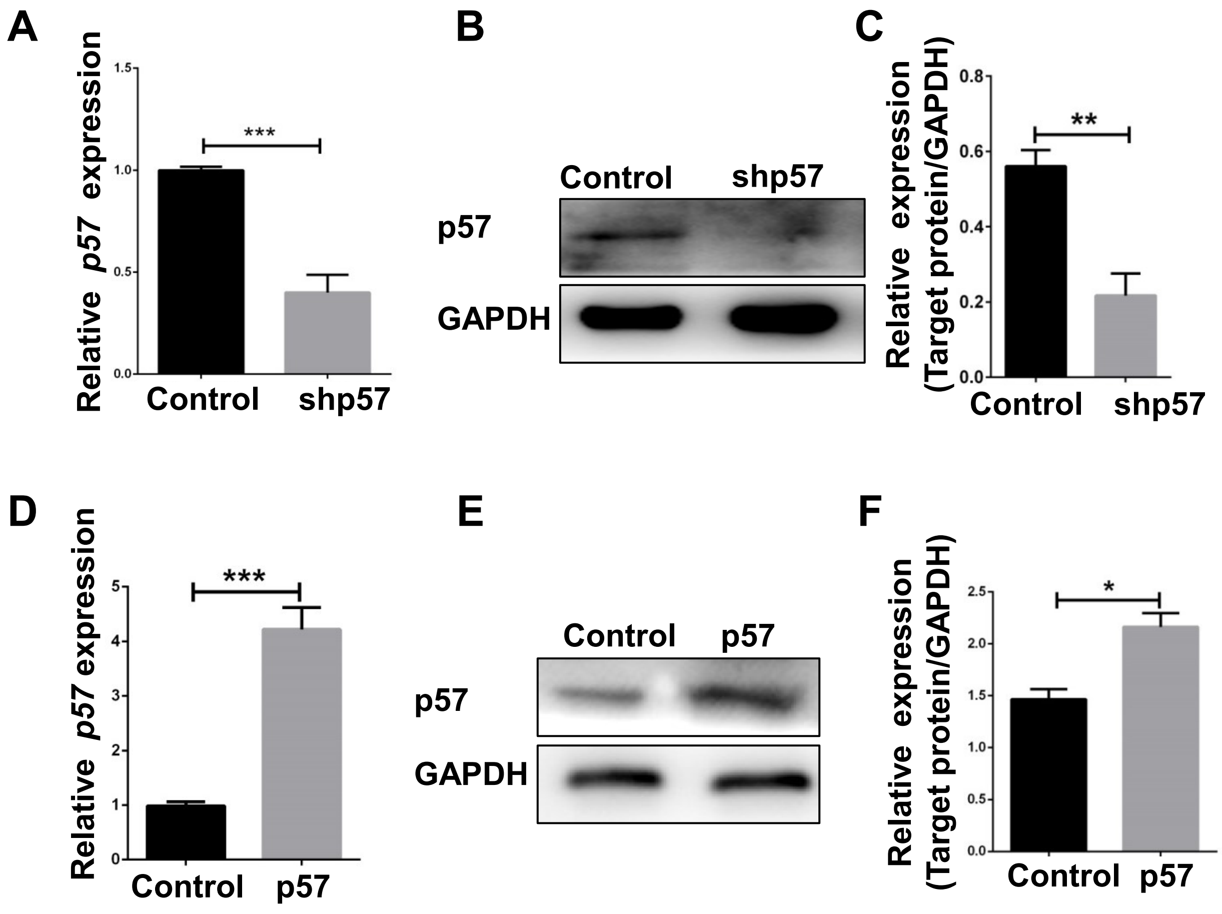

Supplementary Figure 3

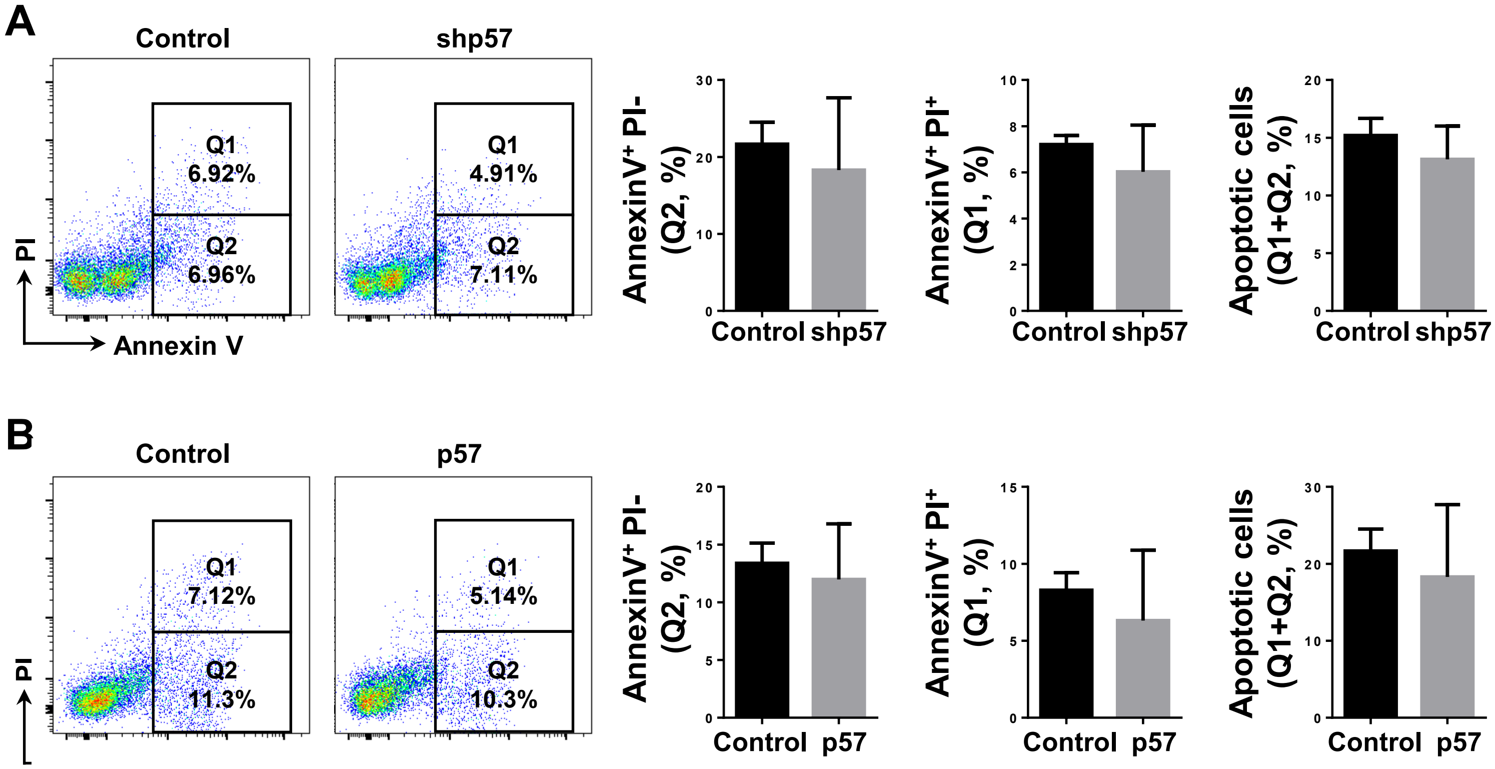

Supplementary Figure 4

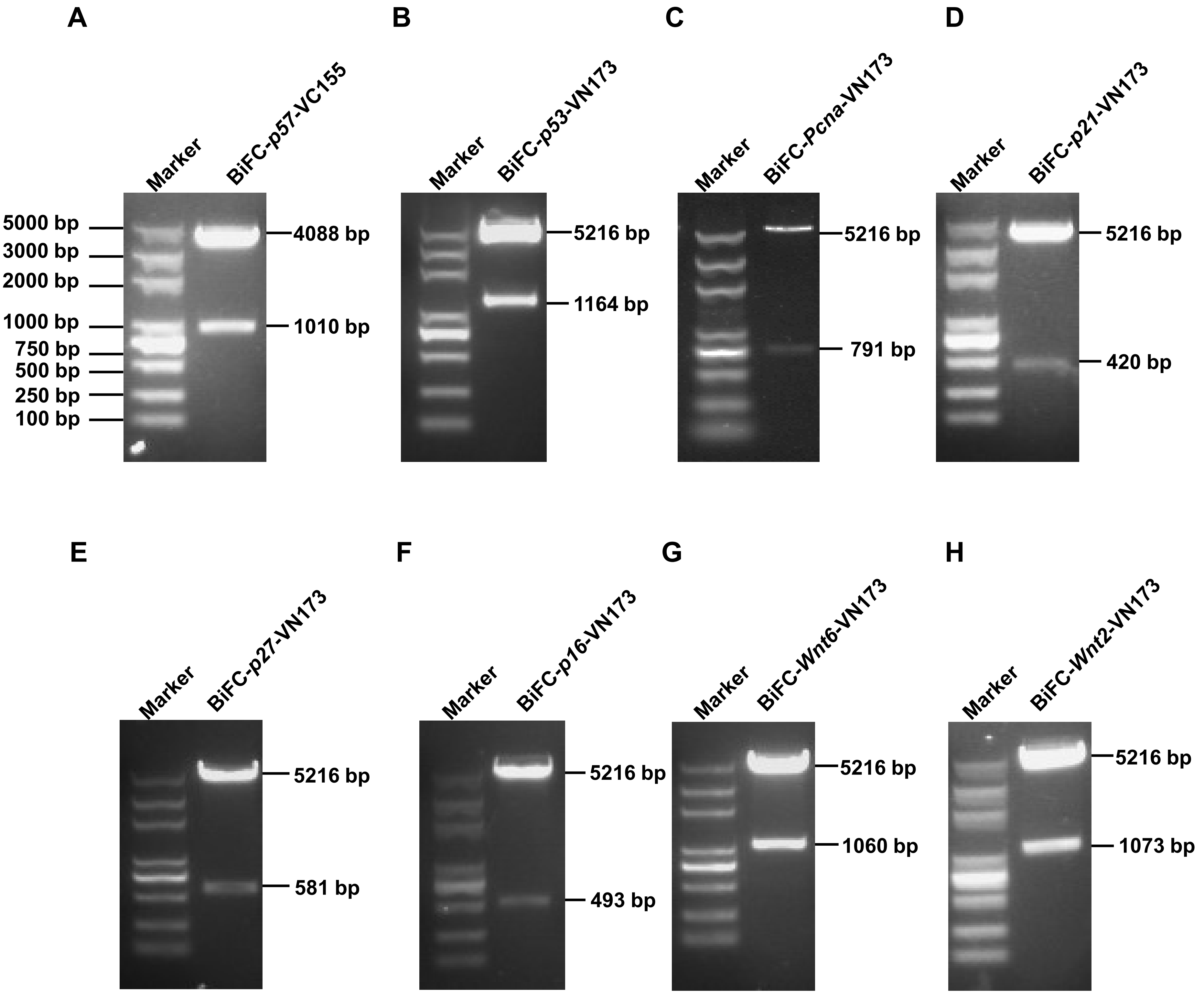

Supplementary Figure 5

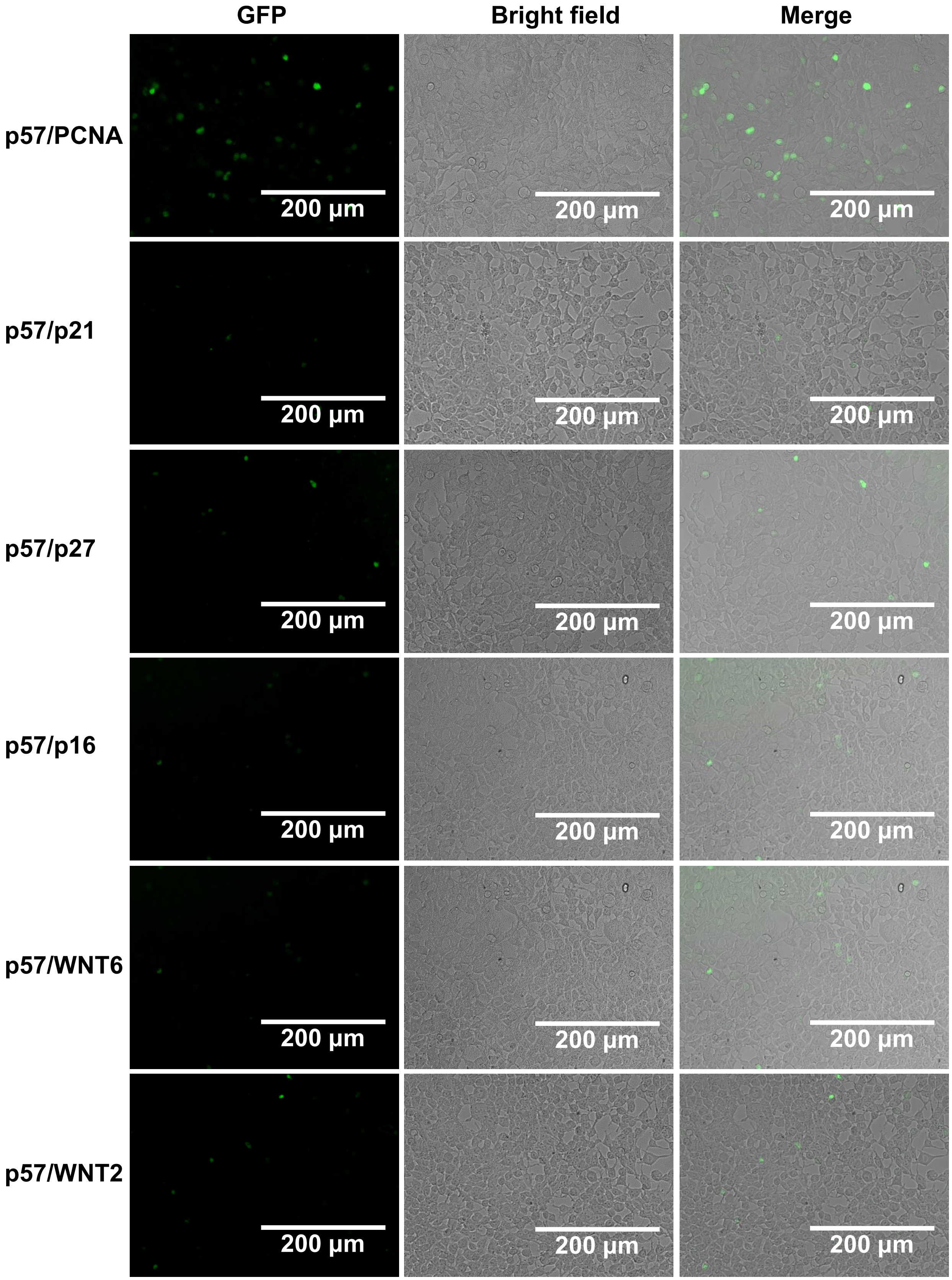

Supplementary Figure 6

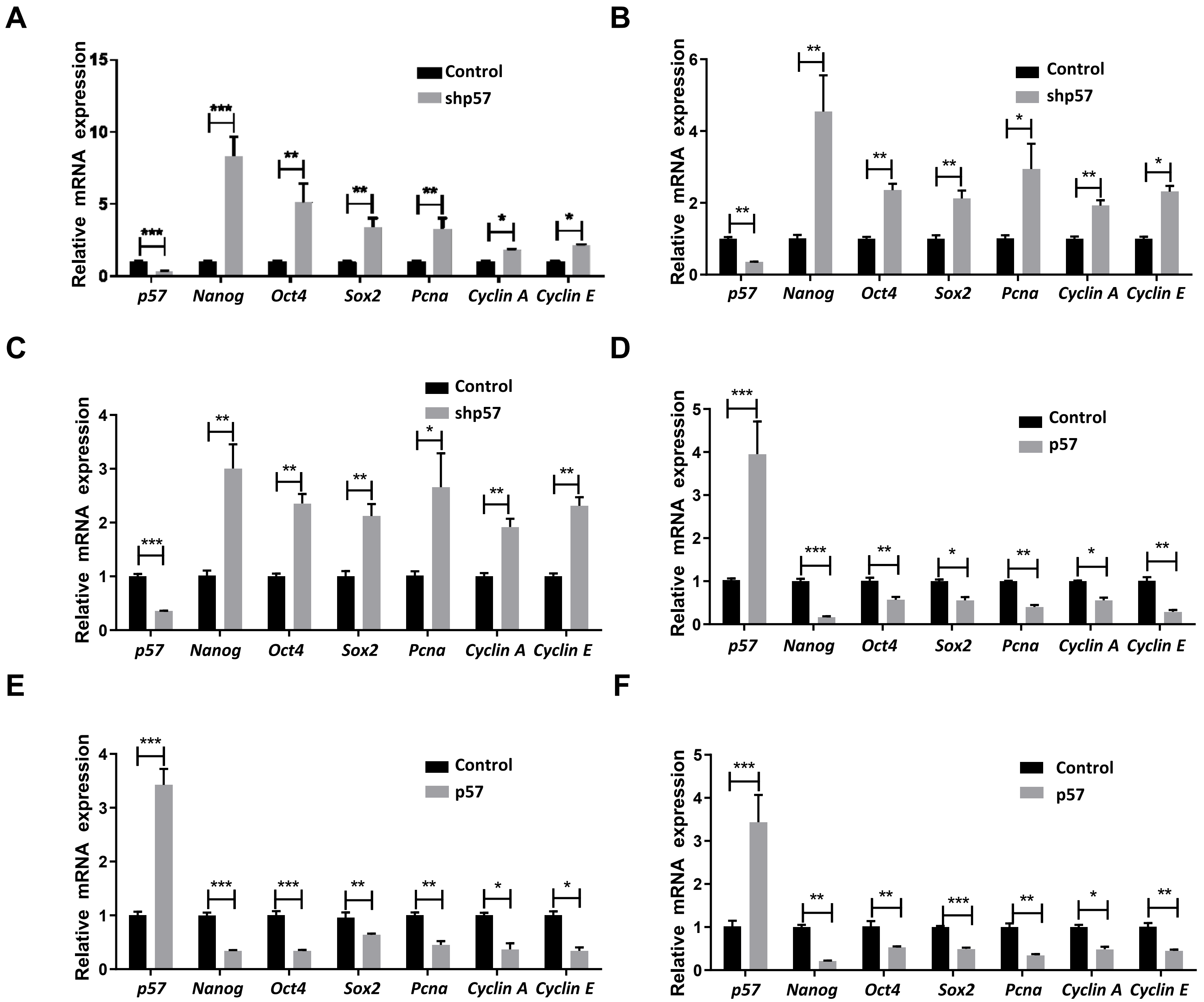

Supplementary Figure 7

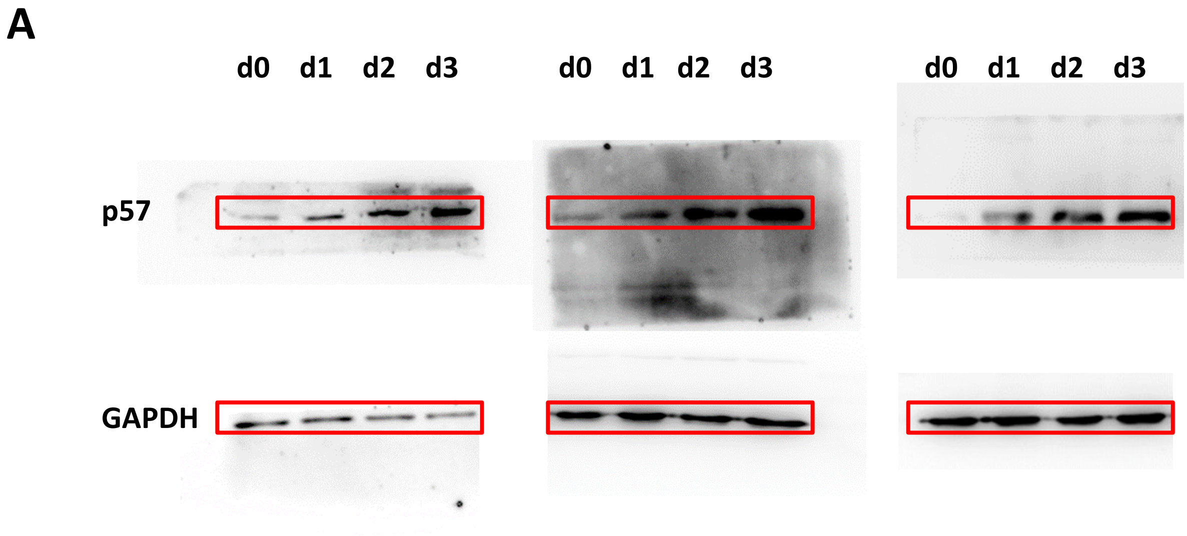

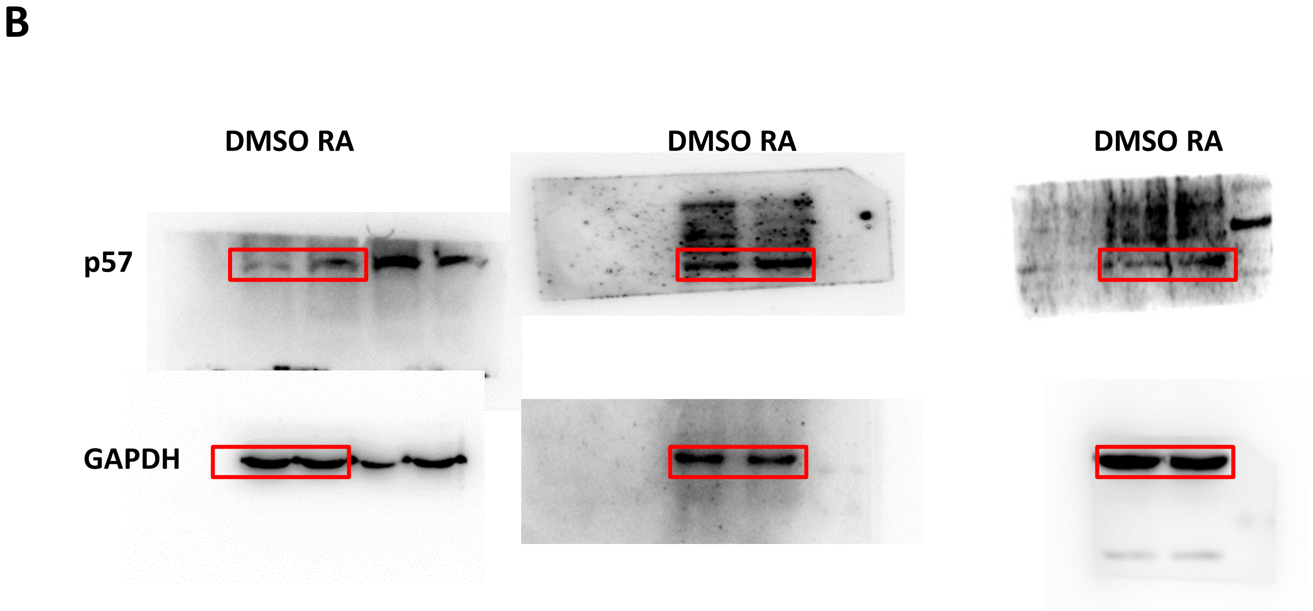

Supplementary Figure 8

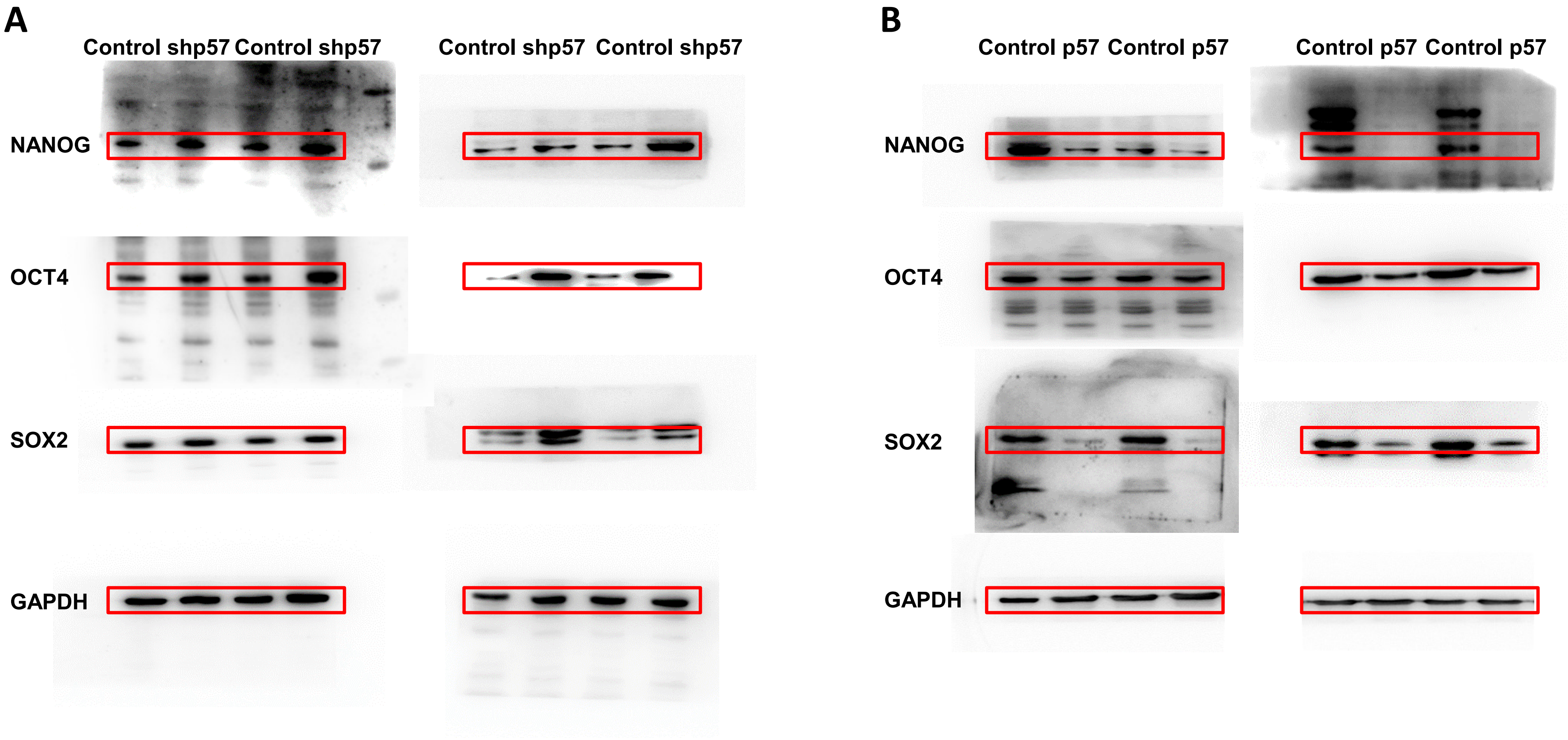

Supplementary Figure 9

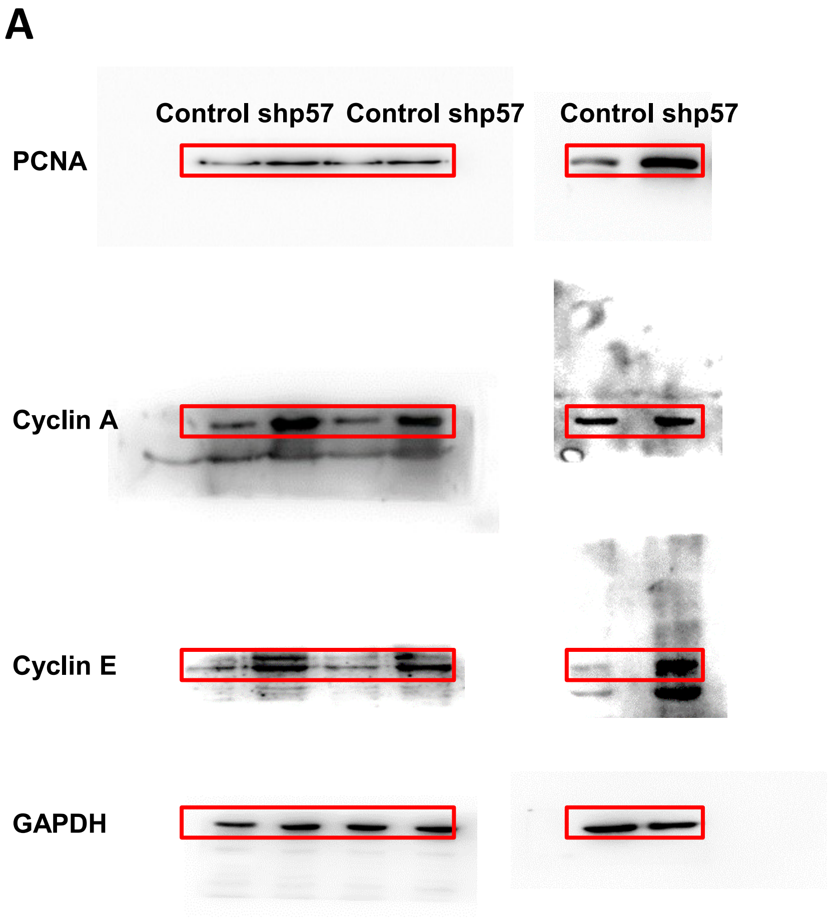

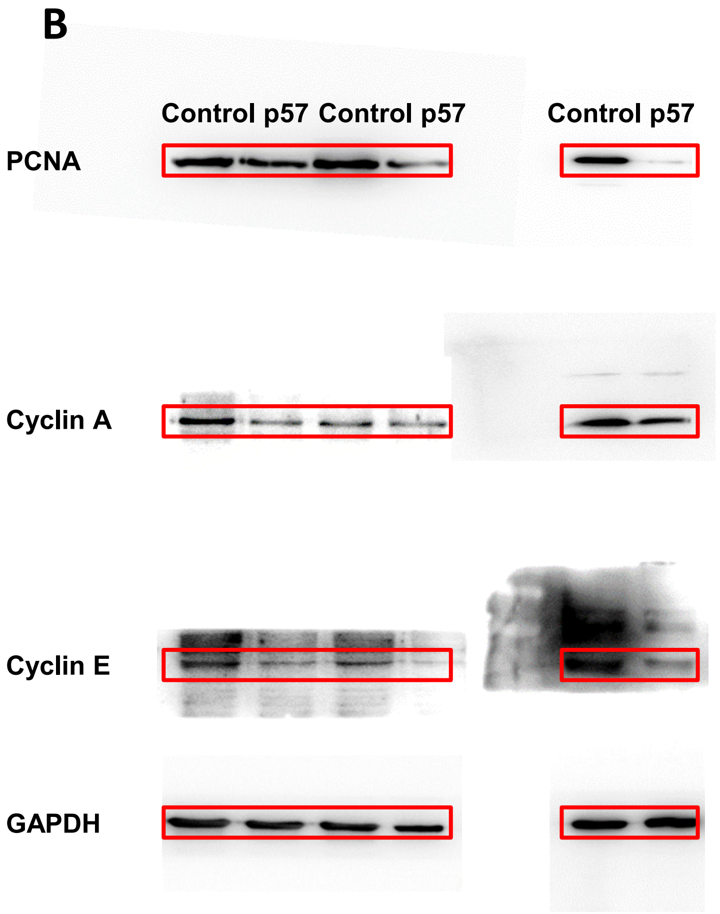

Supplementary Figure 10

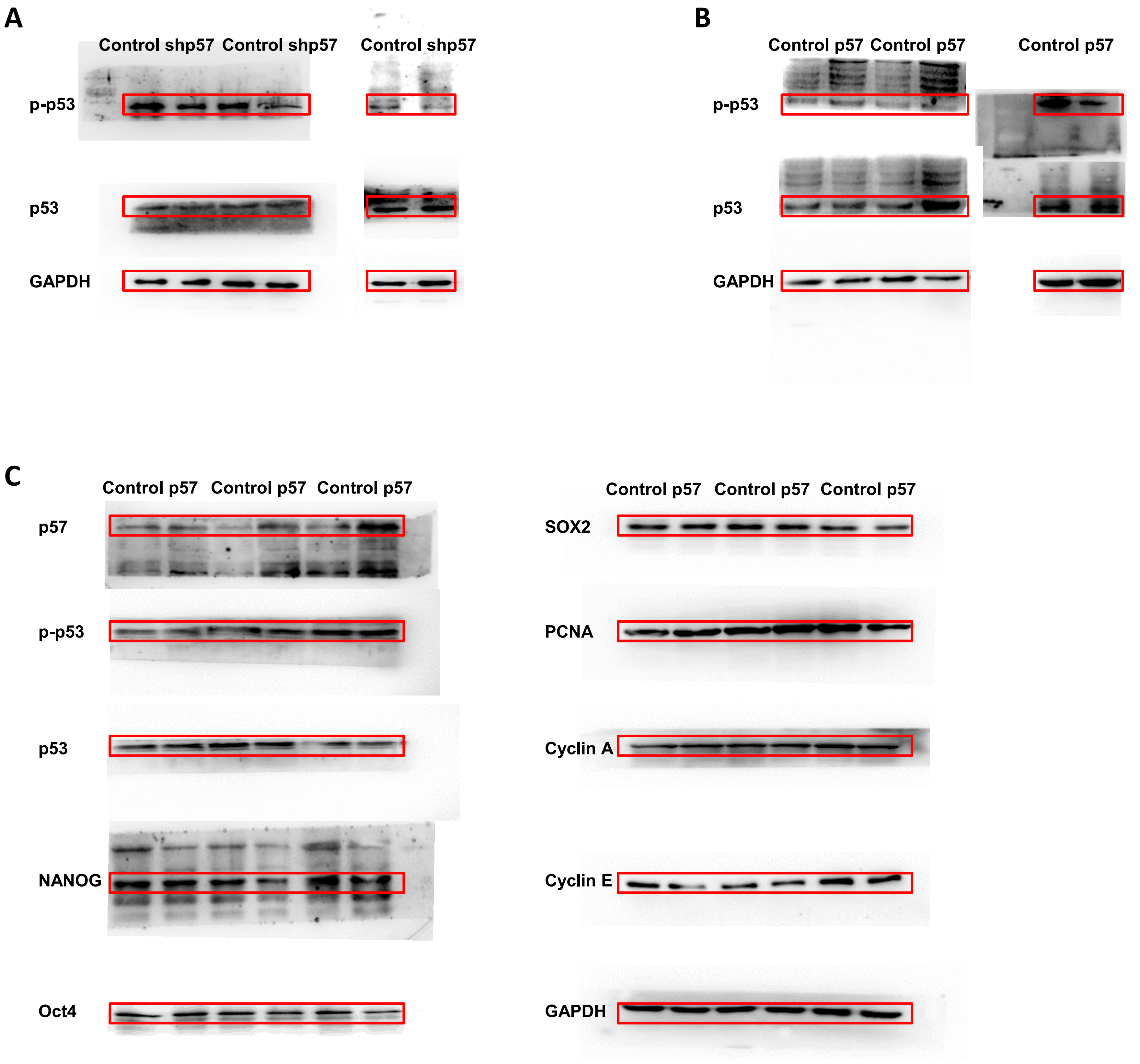

Supplementary Figure 11

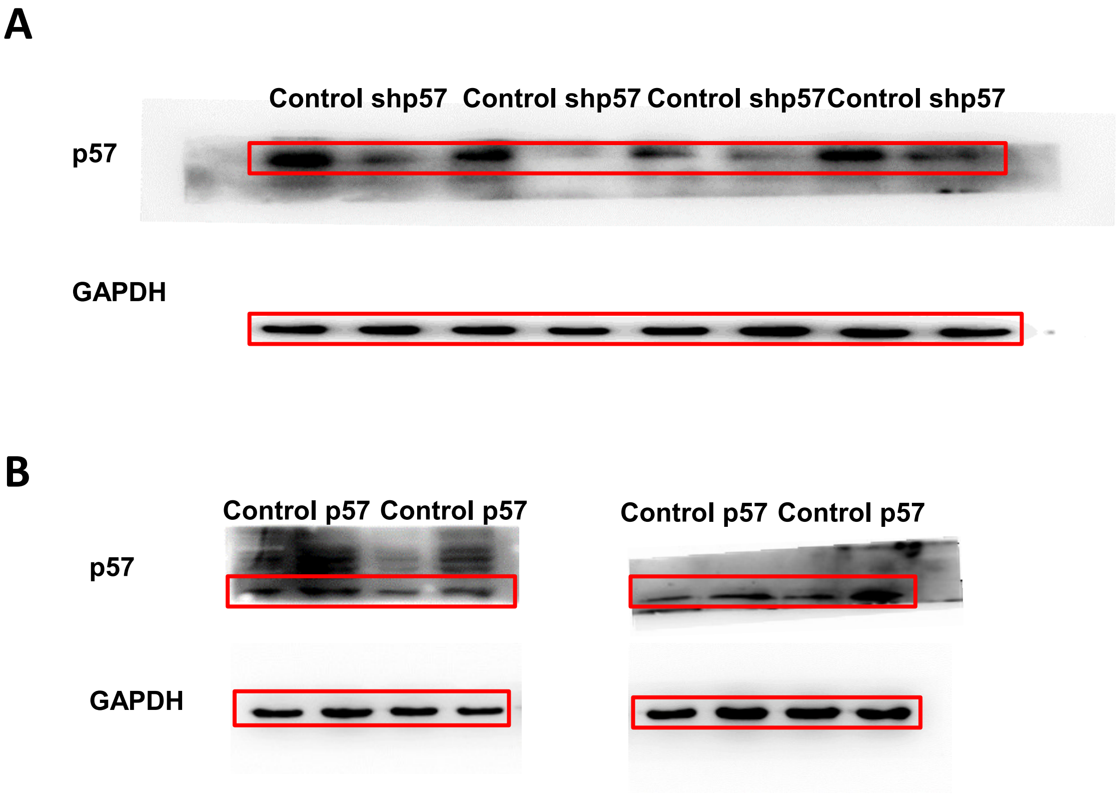

Supplement: Supplementary Materials — Table S1: primer sequences used for real-time quantitative PCR. Table S2: primer sequences used for PCR amplification in BiFC assay. Figure S1: construction of shp57- and p57-overexpressing vectors. Figure S2: p57 knockdown or overexpression efficiency in ESCs. Figure S3: p57 have no effects on apoptosis of mESCs. Figure S4: construction of the vectors for BiFC assay. Figure S5: visualization of the interactions between p57 and candidate proteins (PCNA, p21, p27, p16, WNT6, and WNT2) in vivo by BiFC assay. Figure S6: the effect of p57 on mESCs at day 2, day 4, and day 6. Figure S7: increased p57 protein expression during mESC differentiation. Figure S8: p57 suppressed the pluripotency state of mESCs. Figure S9: p57 suppressed the proliferation of mESCs. Figure S10: p57 interacted with and contributed to the activations of p53 in mESCs. Figure S11: p57 knockdown or overexpression efficiency in mESCs. [file 4968649.f1.zip › Supplementary Figures (2).pdf]
