# Supplementary material for: p57 Suppresses the Pluripotency and Proliferation of Mouse Embryonic Stem Cells by Positively Regulating p53 Activation
Source: Stem Cells Int. 2021 Dec 24;2021:4968649. doi: 10.1155/2021/4968649 (PMC8720024; doi:10.1155/2021/4968649)
Supplement: Supplementary Materials — Table S1: primer sequences used for real-time quantitative PCR. Table S2: primer sequences used for PCR amplification in BiFC assay. Figure S1: construction of shp57- and p57-overexpressing vectors. Figure S2: p57 knockdown or overexpression efficiency in ESCs. Figure S3: p57 have no effects on apoptosis of mESCs. Figure S4: construction of the vectors for BiFC assay. Figure S5: visualization of the interactions between p57 and candidate proteins (PCNA, p21, p27, p16, WNT6, and WNT2) in vivo by BiFC assay. Figure S6: the effect of p57 on mESCs at day 2, day 4, and day 6. Figure S7: increased p57 protein expression during mESC differentiation. Figure S8: p57 suppressed the pluripotency state of mESCs. Figure S9: p57 suppressed the proliferation of mESCs. Figure S10: p57 interacted with and contributed to the activations of p53 in mESCs. Figure S11: p57 knockdown or overexpression efficiency in mESCs. [file 4968649.f1.zip › Supplementary tables (1).docx]

**Supplemental Table 1**

Primer sequences used for real-time quantitative PCR

|  | **Forward** | **Reverse** |
| --- | --- | --- |
| *Gapdh* | TGGCCTTCCGTGTTCCTAC | GCTTGGCGAAGAAGTCGTT |
| *p57* | GCAGGACGAGAATCAAGAGCA | CTTCAGTCTGTACTTCACT |
| *Pcna* | TTTGAGGCACGCCTGATCC | GGAGACGTGAGACGAGTCCAT |
| *Cyclin A* | TGGCTGTGAACTACATTGA | ACAAACTCTGCTACTTCTGG |
| *Cyclin E* | GTGGCTCCGACCTTTCAGTC | CACAGTCTTGTCAATCTTGGCA |
| *Nanog* | TTCTTGCTTACAAGGGTCTGC | AGAGGAAGGGCGAGGAGA |
| *Oct4* | GGCGTTCTCTTTGGAAAGGTGTTC | CTCGAACCACATCCTTCTCT |
| *Sox2* | GCGGAGTGGAAACTTTTGTCC | CGGGAAGCGTGTACTTATCCTT |

**Supplemental Table 2**

Primer sequences used for PCR amplification in BiFC assay

|  | **Forward** | | **Reverse** |
| --- | --- | --- | --- |
| *p57* | | ATAGCTAGCGCTAGGCCCGACTGAGAGCAAGCGA | GCAGAATTCCTGCTCTACGCAACCATCTCCGGTT |
| *p53* | | ATAGAATTCGACTGCCATGGAGGAGTCAC | ATTTCTAGAGAGTCAGGCCCCACTTTCTTG |
| *Pcna* | | TCTAAGCTTGATCCGCCACCATGTTTGAGGCAC | CAGTCTAGATGCTTCCTCATCTTCAATCTTG |
| *P16* | | ACGGAATTCTGGAGTCCGCTGCAGACAGAC | ATTGGTACCTTGGGATTGGCCGCGAAGTCCC |
| *P27* | | CCGGAATTCTGTCAAACGTGAGAGTGTCTAA | AATGGTACCTCGAAGGCCGGGCTTCTTG |
| *P21* | | GCGGAATTCGTCCAATCCTGGTGATGTCC | TATGGTACCCTGGTCTGCCTCCGTTTTCGGCC |
| *Wnt6* | | AATGAATTCCCTCCCGCCTCGGACTGCTGCT | TCAGGTACCGAGGCACAGGCTGAGTTCCTTG |
| *Wnt2* | | CGCAAGCTTATGAACGTCCCTCTCGGTGGAA | TTAGGTACCGTCGCCCAGTCGGCACTCTTGG |
